# Development and initial validation of the trait and state Mindful Eating Behaviour Scales

**DOI:** 10.1007/s40519-023-01614-8

**Published:** 2023-10-25

**Authors:** Michail Mantzios

**Affiliations:** College of Psychology, Birmingham City University, Room C332, The Curzon Building, 4 Cardigan St, Birmingham, B4 7BD UK

**Keywords:** Mindful Eating Behaviour Scale-Trait, Mindful Eating Behaviour Scale-State, Mindful eating behaviour, Mindful eating, Eating behaviours

## Abstract

**Purpose:**

The quantitative assessment of mindful eating has been challenging, even with the latest additions to the field of multifactorial mindful eating psychometric tools. This manuscript presents the development, validity and reliability assessment of a trait and state Mindful Eating Behaviour Scale across four studies driven by recent theory (Mantzios in Nutr Health 27: 1–5, 2021).

**Methods:**

Study 1 assessed the content validity of the scale through ratings of clinical and research experts in the field. Study 2 inspected the scale through exploratory and confirmatory factor, parallel, correlation, and reliability analyses. Study 3 assessed the temporal stability through a test–retest in a 2-week interval. Study 4 assessed the scale in a randomized control experimental design, where a mindful eating (vs. control group) received the trait scale before consuming chocolate, and an equivalent state scale was modified to assess state changes during the 10-min eating session.

**Results:**

Study 1 yielded items to be reflective and concise of the definition of mindful eating behaviour. Study 2 indicated 2 potential factors through exploratory factor analyses, which were further verified through a parallel analysis, while subscales correlation indicated one-dimensionality, which was further verified through confirmatory factor analysis. In addition, the internal consistency of the scale and subscales was good. Study 3 certified the reliability of the scale over time, while Study 4 indicated that both the trait and state scales were significant indicators of eating mindfully.

**Conclusions:**

Together, all studies signal the utility of theoretically sound and empirically validated measurements for the replicable assessment of mindful eating behaviour.

*Level of evidence*: No level of evidence: basic science.

**Supplementary Information:**

The online version contains supplementary material available at 10.1007/s40519-023-01614-8.

## Introduction

Mindful eating has emerged as an effective method for managing appetite, addressing problematic eating patterns, and fostering a balanced and enjoyable approach to food. Mindful eating was motivated by secular mindfulness practices that are descriptive of being attentive to the present moment while maintaining an awareness that entails a non-judgmental attitude [e.g., 1]. Despite research showing positive behaviour changes from mindful eating [2, 3], the subjective definitions, practices, and measurements of mindful eating challenged exact clinical and non-clinical assessments and healthcare professional advice.

Several examples indicate how psychometric measurements may pose problems for future practical research and clinical advice. For example, Framson et al. [4] proposed a Mindful Eating Questionnaire (MEQ) that, according to Hulbert-Williams et al. [5], was criticized for not adequately measuring components of mindfulness. In response, Hulbert-Williams et al. [5] developed a Mindful Eating Scale (MES), which was more aligned with mindfulness theories. Winkens et al. [6] argued that this still did not fully reflect those theories and practices and proposed the Mindful Eating Behavior Scale (MEBS), which diverged from traditional descriptions of secular mindfulness practices. In addition, the MEBS measuring decision-making before and during eating, deviated from a purely behavioural scale [7, 8]. Later analyses indicated that the narrow interpretation of mindfulness and mindful eating (i.e., the focused attentional aspect of mindful eating) did not encompass all aspects that should be considered [9, 10].

Peitz et al. [9] responded to the limitations of the MEBS by validating the Mindful Eating Inventory (MEI). The MEI includes subscales that assess the relationship between the earth, all living beings, and eating, which may present an overly speculative interpretation of mindful eating, thus also diverging from the literature on secular mindfulness. Carrière et al. [10] provided a more detailed account of the limitations of the MEI to justify the development and validation of the Four Facet Mindful Eating Scale (FFaMES). However, the FFaMES includes items, such as ‘My emotions control what I eat’ and ‘My emotions control how much I eat’, which clearly reflect emotional eating and resemble the limitations of Framson et al.’s MEQ [7, 8]. Both the MEQ and FFaMES propose psychometric tools that are influenced by associations with eating behaviours, such as emotional eating. Yet, these mindful eating scales overlooked motives to start eating, such as ‘social’ and ‘conformity’ motives [11], and motives to stop eating, such as ‘decreased food appeal’ and ‘planned amount’ [12, 13]. Therefore, the motives of hunger and satiety with mindful eating behaviour ought to be explored independently, as there are more motives that predict when and how much people eat. Before the emergence of mindful eating psychometric tools, hunger and satiety were independent and separate fields of research, and past findings did indeed suggest a positive impact on eating [14, 15]. Hence, mindful eating psychometric tools go beyond solely focusing on the impact of mindful eating and incorporate elements, such as listening to hunger and satiety and overcoming emotional eating. Overall, and despite the positive contribution to the development of thought and inquiry, existing psychometric tools hinder the development of cost-effective or easily implemented practices.

The nature and limitations of existing mindful eating psychometric tools may have, at least partially, contributed to mixed findings. For example, mindful eating sub-scales have related differently to disordered eating (i.e., positive and negative relationships) [16]. Furthermore, mindful eating showed no relationship to mindfulness, and only in some studies related to self-compassion [17–19] when there is an expectation for mindful eating to consistently relate to both mindfulness and self-compassion. Mantzios [7, 8] highlighted the need for simplicity in the field, arguing for the need for aligning principles of secular mindfulness practice when defining mindful eating. For example, by separating mindful eating behaviour (i.e., sensory experience of eating, non-judgmentally) from decision-making for mindful eating (i.e., “am I still hungry?” “I will not multitask while eating”), so that this is simple and clear. As in mindfulness meditation, the planning of where, when and how to meditate is not assessed in mindfulness psychometric tools, so it should not be for mindful eating. Mantzios further proposed a definition for *Mindful Eating Behaviour* (MEB), described as *“*the sustained attention to a sensory element of the eating experience (e.g., the taste) and a non-judgmental (or non-evaluative) awareness of thoughts and feelings that are incongruent to the sensory elements of the present eating experience” (p. 369) [7]. The proposed definition offers a more rigorous and precise foundation for testing, building on empirical evidence that can be replicated. Developing a scale that aligns with this definition is an important step in assessing mindfulness and mindful eating practices and investigating their associations with other eating behaviours and overall well-being.

### Present research

The present manuscript outlines four separate studies. First, experts were asked to evaluate the items of a newly developed scale while reflecting on the definition of mindful eating behaviour. Second, the factorial structure of the scale and internal consistency were examined. Furthermore, associations between mindful eating, eating behaviours, and mindfulness were assessed to validate the scale. Third, stability over time was assessed by administering the scale twice over a 2-week interval. Fourth, a randomized controlled experiment was conducted to investigate the effectiveness of both the trait and state versions of the Mindful Eating Behaviour Scale in a single-session experimental design, and to compare a mindful eating practice group to a control group.

## Study 1: Expert inter-rater content validity

### Participants

Expert participants were researchers (*n* = 13) and clinical staff (*n* = 18) working with mindful eating. The clinical staff sample comprised practising psychologists in the National Health Service (NHS) (*n* = 6), dieticians/nutritionists (*n* = 3), and nurses (*n* = 9). The overall sample of 31 expert reviewers consisted of 11 males, 18 females and 2 who chose not to disclose their gender. The ethnical/racial background was as follows: white (*n* = 29), Asian (*n* = 1), and one participant who did not provide a response, and with a mean age of 40.7 (*SD* = 11.3). Expert participants were recruited through Prolific, a research participation platform that reimburses participants for their time (£6.00/h). Researchers in the area of mindfulness and mindful eating were approached independently via email and were reimbursed through Amazon vouchers for their time and feedback.

### Procedure

Participants were invited to fill in the expert evaluation online. They were provided with participant and consent forms, and upon consenting were presented with demographic questions. Next, the experts viewed the definition of mindful eating behaviour (see the introduction, Mantzios [7]). They were asked to reflect on this definition while assessing the items. For each item, there were four questions: “1. How relevant is this item to the definition of mindful eating behaviour provided?”, “2. How clear and concise is this item?”, “3. How well does this item reflect the dimension of “sensory attention”?”, and “4. How well does this item reflect the dimension of “non-judgmental awareness”?”. The response range on a Likert scale was 1 (“very poor”) to 5 (“very good”).

### Data analysis

Results were analysed through the Aiken’s *V* formula, which showed non-significant differences between clinicians and researchers. To calculate content validity, Aiken’s *V* formula enabled a content-validity coefficient that was based on the outcomes of the experts’ item rating, with a *V* value above 0.65 indicating retention of the item and adequate content validity [20].

### Results

Experts’ ratings proposed adequate Aiken’s *V* values (i.e., > 0.65, lowest values 0.78) that ensured that there was acceptable content validity to proceed to further studies on the standardisation of the scale and inferential statistics (see Table 1).

**Table 1:** Aiken’s *V* values obtained from expert reviewers’ ratings for Mindful Eating Behaviour Scale-Trait

	Expert evaluation			
	Relevance	Clarity	“Sensory attention” relevance	“Non-judgmental awareness” relevance
Item 1	.90	.81	.92	
Item 2	.93	.91	.95	
Item 3	.90	.92	.88	
Item 4	.89	.88	.91	
Item 5	.87	.82	.78	
Item 6	.90	.86		.87
Item 7	.85	.87		.80
Item 8	.82	.90		.88
Item 9	.83	.80		.83
Item 10	.87	.86		.85

## Study 2: Exploratory factor analysis, internal consistency and convergent validity assessments

### Participants

The sample consisted of 301 participants (180 female, 1 undisclosed), with a mean age of 41.0 (SD = 11.9) and a mean BMI of 29.8 (SD = 8.6). The majority reported to be omnivores (*n* = 283), and others reported to follow a vegetarian diet (*n* = 8), vegan diet (*n* = 2), and non-disclosed information (*n* = 8). Most participants were White (*n* = 273), followed by smaller groups of Black (*n* = 7), Asian (*n* = 12), and mixed-race (*n* = 7) participants (2 participants did not disclose any background). Subject-to-variables ratio was assessed for the recruitment of adequate participants, where an acceptable 8 to 1 [21] was exceeded bearing in mind the validity testing that was also conducted on the same sample. With significance set at 0.05, medium effect size, power = 0.80, and 5 variables in one model, indicated a sample size of 126 participants [22, 23]. To ensure that consideration would be given to average and obese populations, the recruitment focused on an average-weight population until reaching half of the sample, and the rest specifically targeted obese populations. An ability to identify individual differences and develop a scale that would be applicable across different subsamples led to separate identical analyses depending on BMI categorisation. Exclusion criteria were self-determined by the participant and included any current mental health or eating disorder diagnoses, and age under 18.

### Materials

*The Mindful Eating Behaviour Scale-Trait* (*MEBS-T*) [24]. The scale contains of 10 items that measure two components of mindful eating behaviour: “Sensory Attention” (5 items) and “Non-judgmental Awareness” (5 items). Both factors are descriptive and align with mindful eating behaviour and mindfulness theory. Sample questions include “I fully taste what I am eating” and “I hold my attention on what I am eating, despite recognising the occurrence of thoughts and/or feelings while I am eating”. The scale utilises a 4-point Likert scale with responses ranging from 1 (*strongly disagree*) to 4 (*strongly agree*). Internal consistency for the total score of the scale was good (*α* = 0.85), and similarly for the subscales “Sensory Attention” (*α* = 0.85) and “Non-judgmental Awareness” (*α* = 0.82).

*Five-Facet Mindfulness Questionnaire-15* (*FFMQ-15*) [25]. This is a shorter version of the original 39-item FFMQ, which measures five facets of mindfulness: *Observing, Describing, Acting with Awareness, Non-Judging* and *Non-Reactivity* (each being 3 items). Sample questions include “I do jobs or tasks automatically without being aware of what I’m doing” and “I find myself doing things without paying attention”. Responses are recorded on a 5-point Likert scale ranging from 1 (*never or very rarely*) to 5 (*very often or always true*). A score is combined for each facet of the scale. Internal consistency for the overall score was acceptable (*α* = 0.79), and, similarly for *describing* (*α* = 0.81), *acting with awareness* (*α* = 0.78), *non-judging* (*α* = 0.85) and *non-reactivity* (*α* = 0.72), while for *observing* the value was lower than indicated for cutoff values (*α* = 0.65).

*Dutch Eating Behaviour Questionnaire* (*DEBQ*) [26]. The DEBQ is a 33-item scale containing items in *External* (*DEBQ-ExE*; 10 items), *Restrained* (*DEBQ-RE*; 10 items) and *Emotional Eating* (*DEBQ-EE*; 12 items; note that item 28 is not part of the three main subscales). Sample items include “Do you have the desire to eat when you are irritated?” and “Do you have a desire to eat when you have nothing to do?”. Responses are recorded on a 5-point Likert scale on a 5-point Likert scale ranging from 1 (*never*) to 5 (*very often*). Internal consistency for the present research was good for External Eating (*α* = 0.88), Restrained Eating (*α* = 0.89), and Emotional Eating (*α* = 0.96).

*Grazing Scale* (*GS*) [27]. The 8-item Grazing Scale investigates the repetitive eating of small amounts of food. A sample item is ‘Have you ever felt compelled or driven to eat, even when not hungry?’, and responses range from 1 (*rarely*) to 5 (*all of the time*). Internal consistency for the present research was good (*α* = 0.92).

### Procedure

Participants were provided with a link to an online platform (Prolific), and were reimbursed for their participation time (£6.00/h). Participants first viewed the participant information and consent forms, and upon consenting were presented with the demographics page and the psychometric material (i.e., MEBS-T, FFMQ-15, GS, and DEBQ). The exact opposite order (i.e., DEBQ, GS, FFMQ-15, and MEBS-T) was administered for half of the participants to counterweigh the administration of psychometric tools. The order of administration did not impact on any differences. After completing the materials, the participants were directed to a debriefing page before their participation concluded.

### Data analysis

Data screening was conducted prior to inferential analyses to evaluate whether assumptions were met regarding the presence of outliers, multivariate normality, linearity, and homogeneity of variance. The Kaiser–Meyer–Olkin (KMO) measure of sampling adequacy and Bartlett’s test of sphericity were also evaluated preceding any attempts to conduct exploratory factor analyses to ensure data fitness. Once all assumptions were satisfied, Exploratory Factor Analysis (EFA), with principal axis factor extraction and oblique rotation was performed. Screeplot identification, eigenvalue (> 1), and higher item loading greater than 0.30 were criteria to evaluate factor extraction, with the addition of a Monte Carlo PCA for parallel analysis indicative of rejecting or accepting factors [28, 29]. Once the factor structure was identified, Pearson’s correlations between the subscales were performed to investigate the potential of an overall score calculation for the scale, as well as Cronbach’s *α* internal consistency coefficients were calculated for both the overall scale and subscales. All data analyses to this point were analysed using IBM SPSS 28.

Data were further analysed using AMOS 24. “Sensory Attention” and “Non-judgmental Awareness” were first-order latent factors that loaded onto a second-order latent factor; that is, “Mindful Eating Behaviour Scale”. Structural equation modelling was run using the maximum-likelihood method, and Confirmatory Factor Analysis (CFA) goodness-of-fit was assessed for this one factor, second-order model, which included indexes of fit: a Chi-squared by the degree of freedom (*χ*^2^ CMIN/df) ratio < 5; root mean square error of approximation (RMSEA) < 0.08; Adjusted Goodness of Fit Index (AGFI), the Goodness of Fit Index (GFI), Tucker–Lewis Index (TLI), Comparative Fit Index (CFI), and Incremental Fit Index (IFI) > 0.9; Parsimony Normed Fit Index (PNFI) > 0.5 [30–32].

### Results

The acceptability of the factorial structures was assessed by exploring the Kaiser–Meyer–Olkin (KMO) and the Bartlett’s sphericity test. The KMO measure of sampling adequacy was 0.85, and Bartlett’s test of sphericity (*p* < 0.001) indicated that the assumptions for a factor analysis were met. Principal component analysis revealed the presence of two components with eigenvalues > 1, explaining 42.9% and 19.2% of the variance. An inspection of the screeplot revealed a break after the second component, highlighting the items that loaded on the two subscales. Parallel analysis showed that both components should be accepted (see Table 2). Oblimin rotation was performed assuming that there would be an overall correlation between subscales as all the items were set to reflect and measure mindful eating behaviour. The analysis showed strong loadings (> 0.5), apart from items 5 and 7. The two-factor solution explained a total of 62.2% of the variance. The sample was separated, and isolated factorial analyses were repeated for obese and average-weight subsamples. While the findings were analogous between the overall sample and the obese sample, the average weight (BMI < 30) identified items 5 and 7 to load onto a third factor (eigenvalue = 1.007). This violated the theoretical assumption of the two-factor structure of the scale. Thus, items were excluded from the final version of the scale to ensure consistency of comparisons between groups and clarity in identifying individual differences in future research. Correlations between subscales were significant and of moderate strength (*r* = 0.42), corresponding to the association that would be expected in a homogeneous scale (Table 3).

**Table 2:** Comparison of eigenvalues from principal components analysis (PCA) and the corresponding criterion values obtained from parallel analysis for Mindful Eating Behaviour Scale-Trait

Component number	Eigenvalue from PCA	Parallel analysis value	Decision
1	4.295	1.294	Accept
2	1.924	1.204	Accept

**Table 3:** Factor loadings for exploratory analysis with oblique rotation of Mindful Eating Behaviour Scale-Trait

	1	2
Item 1	.917	
Item 2	.809	
Item 3	.783	
Item 4	.854	
Item 5	.474	
Item 6		.789
Item 7		.333
Item 8		.845
Item 9		.900
Item 10		.822

Reliability was assessed by estimating Cronbach’s alpha values for the total score, and the subscales that were specified during the development of the scale. Both the “Sensory Attention” subscale (*α* = 0.85), and the “Non-judgmental Awareness” subscale (*α* = 0.82), as well as the total score (*α* = 0.85), exceeded the recommended values for an internally consistent scale.

Pearson’s correlations were conducted between the MEBS-T, GS, DEBQ, and FFMQ-15 (see Table 4). MEBS-T showed a significant negative correlation to emotional eating and a significant positive correlation to overall mindfulness scores. Interestingly, the Sensory Attention subscale displayed a significant negative relationship to DEBQ-EE and GS, while the Non-judgmental Awareness subscale displayed a positive significant relationship to DEBQ-RE.

**Table 4:** Pearson’s correlations were conducted between the MEBS-T, DEBQ, GS, and FFMQ-15

	1	2	3	4	5	6	7
1.MEBS-T	1						
2.MEBS-T(SA)	.832^**^	1					
3.MEBS-T (NJA)	.852^**^	.418^**^	1				
4.DEBQ-EE	− .141^*^	− .278^**^	.025	1			
5.DEBQ-ExE	.026	− .071	.090	.611^**^	1		
6.DEBQ-RE	.110	.037	.148^*^	.260^**^	.089	1	
7.GS	− .114	− .229^**^	.014	.665^**^	.652^**^	.094	1
8.FFMQ-15	.414^**^	.457^**^	.258^**^	− .357^**^	− .275^**^	− .077	− .404^**^

1. MEBS-T = *The Mindful Eating Behaviour Scale-Trait,* 2. MEBS-T(SA) = *The Mindful Eating Behaviour Scale-Trait,* Sensory Attention*,* 3. MEBS-T (NJA) = *The Mindful Eating Behaviour Scale-Trait,* Non-judgmental Awareness, 4. DEBQ-EE = *Dutch Eating Behaviour Questionnaire,* Emotional Eating, 5. DEBQ-ExE = *Dutch Eating Behaviour Questionnaire,* External Eating, 6. DEBQ-RE = *Dutch Eating Behaviour Questionnaire,* Restrained Eating 7. GS = *Grazing Scale,* 8. FFMQ-15 = *Five-Facet Mindfulness Questionnaire-15*

^**^Correlation is significant at the 0.01; ^*^Correlation is significant at the 0.05

**Fig. 1:**
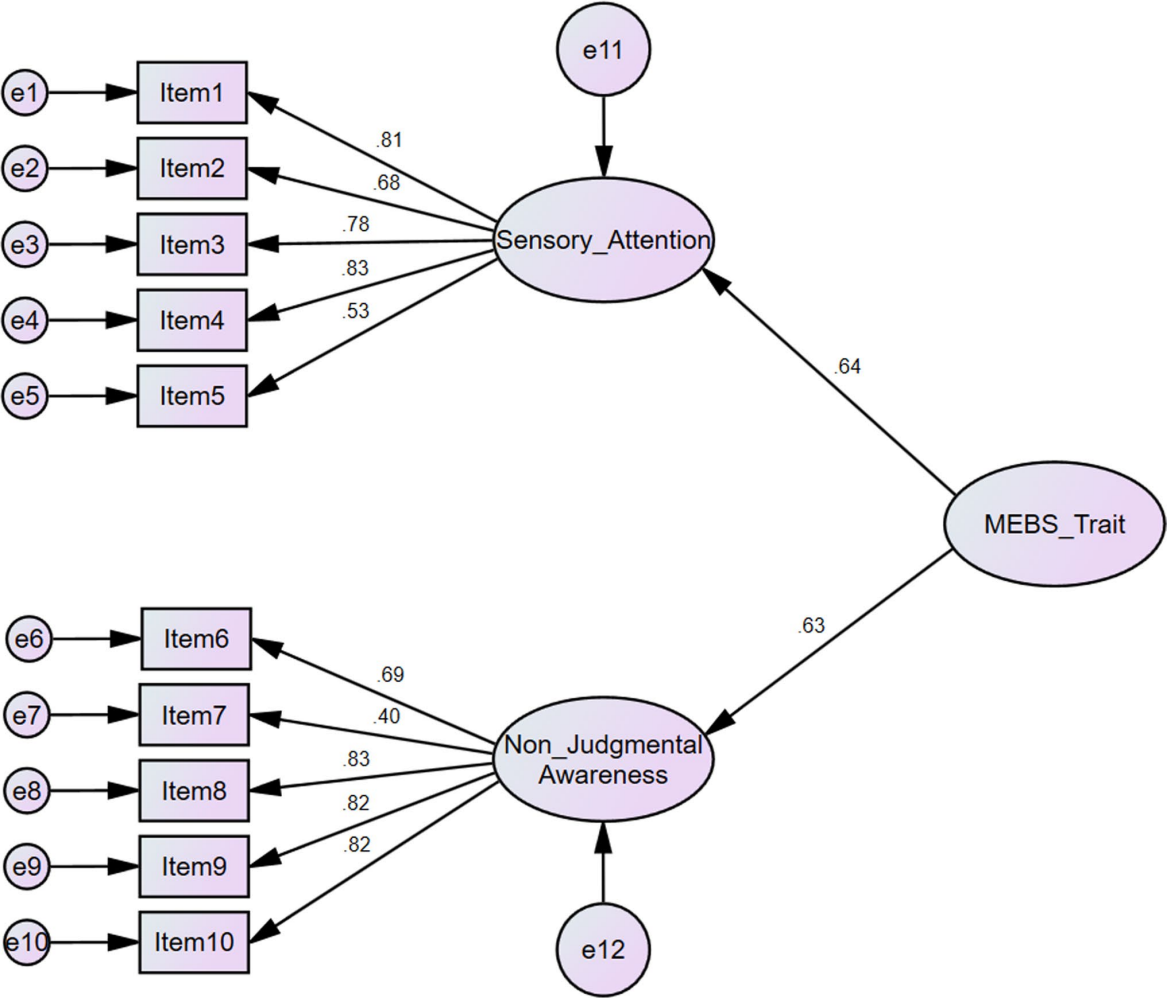
CFA standardised loadings on a second-order single factor of the MEBS-T

The CFA revealed that the 10 items were not a good fit for the proposed model: CMIN/d*f* = 3.75; RMSEA = 0.096; AGFI = 0.87, GFI = 0.92, TLI = 0.91, CFI = 0.93, IFI = 0.93; PNFI = 0.69, despite five of the eight indices of fit indicating an adequate fit. Contrary, the removal of Items 5 and 7, as indicated being the weakest in loading for both the EFA and CFA, proposed a better fit: CMIN/d*f* = 3.32; RMSEA = 0.088; AGFI = 0.90, GFI = 0.95, TLI = 0.94, CFI = 0.96, IFI = 0.96; PNFI = 0.64; all indicating a good fit, apart from the RMSEA marginally exceeding the suggestive value by 0.008 (see Fig. 1 for loadings).

## Study 3: Temporal stability assessment through test–retest reliability

### Participants

The sample consisted of 125 participants (78 female), with a mean age of 46.1 (SD = 13.1) and a mean BMI of 33.4 (SD = 5.0). Most participants were White (*n* = 119), followed by smaller groups of Black (*n* = 2), Asian (*n* = 1), and mixed race (*n* = 2) participants (1 participant did not disclose any background). With significance set at 0.05, medium effect size, power = 0.80, a sample size of 125 participants was recruited [22, 23]. Participants who took part in Study 2 were not invited to take part in this research, via adding them to the exclusion criteria of Prolific for this research study. Other exclusion criteria were self-defined current mental health and eating disorder diagnoses, as well as being under the age of 18.

### Materials

*The Mindful Eating Behaviour Scale-Trait* (*MEBS-T*; Mantzios, 2022). Please see Study 2 for the scale description. The Cronbach’s alpha values in the present study were good for the total score of the scale (*α* = 0.82 at baseline; *α* = 0.90 at retest), and for the subscales “Sensory Attention” (*α* = 0.78 at baseline; *α* = 0.87 at retest) and “Non-judgmental Awareness” (*α* = 0.80 at baseline; (*α* = 0.88 at retest).

### Procedure

Participants were invited to take part in an online two-part study, through Prolific, asking them to complete an eating behaviours questionnaire twice, over a 2-week interval. Participants were reimbursed for their participation for both times (£6.00/h). In both instances, participants were provided with a link to an online platform, where they viewed the participant information and consent forms, and upon consenting, proceeded to the demographics page and the MEBS-T. On completing the MEBS-T, participants were directed to a debriefing page which then concluded participation.

### Data analysis

Test–retest reliability was assessed to show reliability over time of the MEBS-T. Pearson’s correlations were also used for test–retest reliability, and convergent and divergent validity assessments, with strong correlation coefficients estimated over the value of *r* = 0.50 [33]. All data were analysed using IBM SPSS 28.

### Results

Results showed that over 2-week period, there were significant positive strong correlations between Time 1 and Time 2 for the overall score (*r* = 0.81, *p* < 0.01), the “Sensory Attention” subscale (*r* = 0.55, *p* < 0.01), and the “Non-judgmental Awareness” subscale (*r* = 0.61, *p* < 0.01). These results propose good test–retest reliability.

## Study 4: Experimental assessment of MEBS-T and MEBS-S

### Participants

The sample consisted of 133 participants (79 female, 5 undisclosed), with a mean age of 40.3 (SD = 13.0) years and a mean BMI of 35.4 (SD = 7.3). Most participants were White (*n* = 120), followed by smaller groups of Black (*n* = 3), Asian (*n* = 3), mixed race (*n* = 2) participants, and 5 participants who did not disclose this information. Calculations on choosing a suitable sample size led to aiming for 64 participants for each group, to ensure that there would be a medium effect size at 0.80 power and significance set at 0.01 [22, 23]. The experimental group included 66 participants, and the control group included 67 participants. Participants who took part in Study 2 and Study 3 were not invited to take part in this research, through adding them to the exclusion criteria of Prolific. Additional exclusion criteria were any current self-defined mental health or eating disorder diagnoses, as well as being under the age of 18.

### Materials

*The Mindful Eating Behaviour Scale-Trait* (*MEBS-T*) [24]. Please see Study 2 for the scale description. The Cronbach alpha for the total score of the scale in the present study was good (*α* = 0.89), and similarly good for the subscales “Sensory Attention” (*α* = 0.87) and “Non-judgmental Awareness” (*α* = 0.86).

*The Mindful Eating Behaviour Scale-State* (*MEBS-S*) [34]. The state scale version is an adapted form of the MEBS-T used to measure changes in experimental designs or clinical assessments through single-session responses to mindful eating practices. The items and the factorial structure were adapted for the state scale to resemble the trait version of the scale. The scale is attached in Additional file 1: Appendix S1 at the end of the manuscript. The Cronbach’s alpha in the present study for the total score of the scale was good (*α* = 0.90), and so were the values for both subscales, “Sensory Attention” (*α* = 0.88) and “Non-judgmental Awareness” (*α* = 0.89).

### Procedure

Participants were invited to take part in a two-part online study on Prolific, after which they were reimbursed for their participation (for both parts; £6.00/h). Before agreeing to take part in the study, participants were informed that they would need to consume a chocolate bar before the study commenced and that they would need to eat chocolate during the study. Participants first viewed the participant and consent forms, and upon consenting, completed a demographics page and the MEBS-T. On completion of the MEBS-T, participants were randomly allocated to either the experimental or control group. Both groups were then directed to the chocolate that they were asked to bring for this study. Along with a 10-min countdown, a written message was presented on the screen: “At this point, you get 10 min to enjoy your chocolate. Please have your chocolate (ideally a chocolate bar), and after 10 min you will be directed to finish the study. If you finish your chocolate before the 10 min, press the button below to continue.”

The experimental group further received the Mindful Eating Behaviour Practice (MEBP—see Additional file 1) [3]. When participants were ready to proceed, the next page asked them to complete the MEBS-S that reflected on the past 10 min and respond to questions about the brand of the chocolate, the total grams and the grams consumed. Upon completion, participants viewed a debriefing page, which concluded their participation.

### Data analysis

Evaluating the MEBS state version was performed through a one-way ANCOVA (Groups: Control, Experimental) on the MEBS-S while controlling for the MEBS-T. Similar analyses were conducted for the subscales of the MEBS-S, and the time taken to eat, as well as the amount that was consumed, were also explored as outcome variables. All data were analysed using IBM SPSS 28.

### Results

Before analysing the data, to consider participants’ unrestrained choice of chocolate limiting portion size control, an independent sample *t* test was conducted to compare the grams of chocolate that were used, as well as the BMI of participants. The chocolate available to participants in grams was not significantly different for the two groups, *t*(131) = − 0.87, *p* = 0.39, although the experimental group reported slightly higher grams (*M* = 79.2, SD = 58.3) to the Control group (*M* = 69.2, SD = 73.2). The same was true with the BMI between groups,* t*(131) = − 0.68, *p* = 0.50, where the experimental group displayed a slightly higher BMI (*M* = 35.9, SD = 7.4) compared to the Control group (*M* = 35.0, SD = 7.3).

Controlling for MEBS-T scores showed significant differences between the two groups, where the experimental group showed significantly higher overall MEBS-S scores and Sensory Attention subscale scores, when compared to the control group. Compared to the control group, the experimental group further showed marginally non-significant differences in the scores for the Non-judgmental Awareness subscale. The amount of chocolate eaten was not significantly different, but the experimental group took significantly more time to eat the chocolate than the control group. The impact of the MEBS-T as a covariate in the one-way ANCOVA indicated that there was a significant relationship to MEBS-S and the subscales, as well as the amount eaten, but not the time that was taken to finish eating, while monitoring for the independent groups, findings further suggested significant differences between the MEP and the control condition, where the MEP displayed higher scores for the overall score of MEBS-T and the Sensory Attention subscale, as well as more time, was taken to consume the chocolate, while Non-Judgemental Awareness was marginally non-significant, but still higher than the control group (see Table 5).

**Table 5:** Tests of between-subjects effects with covariant the MEBS-T and descriptive statistics

				MEBS-T (Covariate)			Group		
Dependent variable	Condition	*M*	*SD*	*F*	*p*	η^2^	*F*	*p*	η^2^
MEBS-S	Control	29.97	5.70	49.59	**< .001**	.28	5.65	**.02**	.04
	Diary	31.40	4.92						
MEBS-S(SA)	Control	16.08	2.99	46.43	**< .001**	.27	4.28	**.04**	.03
	Diary	16.71	2.56						
MEBS-S (NJA)	Control	13.89	3.56	26.97	**< .001**	.18	3.75	.055	.03
	Diary	14.68	3.00						
Food consumed	Control	53.37	58.69	6.76	**.01**	.05	.65	.42	.005
	Diary	45.23	28.09						
Time eating	Control	530.53	253.35	.76	.38	.006	5.99	**.02**	.05
	Diary	696.52	490.14						

Bold indicating significance. MEBS-T = *The Mindful Eating Behaviour Scale-Trait,* MEBS-S = *The Mindful Eating Behaviour Scale-State,* MEBS-S (SA) = *The Mindful Eating Behaviour Scale-State,* Sensory Attention*,* MEBS-S (NJA) = *The Mindful Eating Behaviour Scale-State,* Non-judgmental Awareness

### Discussion

The aim of the present research was to develop and assess the psychometric properties of a new scale for measuring mindful eating behaviour, address previous limitations, and examine trait and state changes in mindful eating. In Study 1, experts’ ratings supported the content validity of the scale, allowing for further testing and standardization. In Study 2, analyses revealed and further supported the presence of two factors. Loadings of items were as predicted, apart from items 5 and 7 (i.e., “Any thoughts and/or feelings are around the taste of the food I am eating.” and “I am aware of thoughts and/or feelings ‘coming and going’ without feeling troubled whilst eating.”, respectively), which although showing acceptable loadings, did not load as high as the rest of the items. Separate analyses for average and obese weight individuals highlighted the issues with items 5 and 7 (i.e., the presence of a third factor for average-weight participants), indicating the need to exclude them to adhere to the intended two-factor model of mindful eating behaviour. Overall, these findings add to the theoretical and statistical consistency in creating a reflective measure of mindful eating behaviour. Intercorrelations between subscales the overall and subscale constructs were negatively associated with emotional eating and positively associated with mindfulness. The *Sensory Attention* subscale showed negative associations with emotional eating and grazing, while the *Non-Judgmental Awareness* subscale was positively associated with restrained eating.

These findings support and replicate earlier research on emotional eating and grazing [6, 18]. The lack of associations with external eating presents a novel finding [cf. 6, 10], potentially explained by the inclusion of participants with obesity or the focus on mindful eating as a behaviour. The present scale is a potential clinical tool for people who are struggling with obesity and provides an opportunity to explore mindful eating behaviour in clinical settings. Testing stability over time through a test–retest design, the present findings show a strong association between the two points of assessment, and the ability of the scale to remain stable and reliable over time. Finally, the trait version of the MEBS was significant in experimental setting, accounting for the variance explored in mindful eating practices. The equivalent state version of the scale indicated how a significant increase is manifested in a mindful eating practice (over a control group).

One limitation of this research is the reliance on a participant platform that provided financial reimbursement, which may limit the generalizability of the findings. Future research should test the scale with clinical populations seeking treatment and examine outcomes related to weight regulation and health. These are important indicators of the utility of the scale in advancing scientific inquiries about mindful eating.

The present research contributes to the development of the first psychometric tool on mindful eating that measures the behaviour rather than decision-making or other unrelated eating behaviours. The inseparability between behaviour and decision-making observed in other literature on multifactorial scales does serve a purpose for scientific inquiries or clinical applications when assessing mindful eating programs, but not necessarily changes in mindful eating behaviour. The research presented further proposes methods of testing mindful eating in single-sessions of experimental settings through the state version of the scale, as well as cross-sectionally and longitudinally through the trait version of the scale. Both the state and trait versions are simple and reliable tools for clinical and non-clinical research, rooted in key theories of mindfulness and mindful eating.

### Strength and limits

The present research presents the following limitations: the cross-sectional nature of the analysis, the limited samples, and the lack of further cross-cultural and clinical validity. Strengths are the validation of two new Mindful Eating Behaviour Scales for trait and state measurements, the initial validation and the potential for clinical and non-clinical use.

### What is already known on this subject?

Varied definitions of mindful eating led to psychometric tools that lack validity, and alignment to secular mindfulness practices.

### What this study adds?

The study introduced novel measurement tools to assess mindful eating behaviours. By distinguishing between trait and state factors, the study illuminated the nuances of mindful eating behaviours in various research contexts, providing reliable and valid tools for people, researchers and practitioners. The findings have implications for both research and interventions, addressing gaps in existing literature and advancing theoretical frameworks related to mindful eating.

### Supplementary Information

Below is the link to the electronic supplementary material.

**Additional file 1.** Additional Material: Appendix S1.

## Data Availability

Data will be available on request from the corresponding author.
