# Supplementary material for: Development and initial validation of the trait and state Mindful Eating Behaviour Scales
Source: Eat Weight Disord. 2023 Oct 25;28(1):88. doi: 10.1007/s40519-023-01614-8 (PMC10600279; doi:10.1007/s40519-023-01614-8)
Supplement: Supplementary file 1 — Additional file 1. Additional Material: Appendix S1. [file 40519_2023_1614_MOESM1_ESM.pdf]

Additional Material:

Mindful Eating Behaviour Scale – Trait

There is a list of statements below. Please use the rating scale to indicate how well each statement describes your experience with eating and food.

	Strongly Disagree (1)	Disagree (2)	Agree (3)	Strongly Agree (4)
**1. I fully taste what I am eating.**	○	○	○	○
**2. I notice the smell, texture and/or colours of the food I am eating.**	○	○	○	○
**3. I focus on what I am eating.**	○	○	○	○
**4. I fully taste every bite that I am eating.**	○	○	○	○
***5. Any thoughts and/or feelings are around the taste of the food I am eating.**	○	○	○	○
**6. I notice thoughts and/or feelings that are unrelated to my eating, but I redirect my attention to the food and the experience of eating.**	○	○	○	○
***7. I am aware of thoughts and/or feelings 'coming and going' without feeling troubled whilst eating.**	○	○	○	○
**8. When I am eating, I have thoughts and/or feelings, but keep refocusing on the food.**	○	○	○	○
**9. I hold my attention on what I am eating, despite recognising the occurrence of thoughts and/or feelings while I am eating.**	○	○	○	○
**10. When I am eating, I overcome unrelated thoughts and/or feelings by focusing on the food and the sensation of eating.**	○	○	○	○

*Items excluded from the final version of the scale

Add all 8 items for an overall score on Mindful Eating Behaviour. Items 1-4 constitute the Sensory Attention subscale, and items 6, 8-10 constitute the Non-judgmental Awareness subscale.

Mindful Eating Behaviour Scale – State

There is a list of statements below. Please use the rating scale to indicate how well each statement describes your experience with eating and food in the past 10 minutes or for the duration of having the food that you just consumed.

	Strongly Disagree (1)	Disagree (2)	Agree (3)	Strongly Agree (4)
**1. I fully tasted what I was eating.**	○	○	○	○
**2. I noticed the smell, texture and/or colours of the food I was eating.**	○	○	○	○
**3. I focused on what I was eating.**	○	○	○	○
**4. I fully tasted every bite that I was eating.**	○	○	○	○
***5. Any thoughts and/or feelings were around the taste of the food I was eating.**	○	○	○	○
**6. I noticed thoughts and/or feelings that were unrelated to my eating, but I redirected my attention to the food and the experience of eating.**	○	○	○	○
***7. I was aware of thoughts and/or feelings 'coming and going' without feeling troubled whilst eating.**	○	○	○	○
**8. When I was eating, I had thoughts and/or feelings, but kept refocusing on the food.**	○	○	○	○
**9. I held my attention on what I was eating, despite recognising the occurrence of thoughts and/or feelings while I was eating.**	○	○	○	○
**10. When I was eating, I overcame unrelated thoughts and/or feelings by focusing on the food and the sensation of eating.**	○	○	○	○

*Items excluded from the final version of the scale

Add all 8 items for an overall score on Mindful Eating Behaviour. Items 1-4 constitute the Sensory Attention subscale, and items 6, 8-10 constitute the Non-judgmental Awareness subscale. Ensure that you adjust the time in the instructions from "10 minutes" to the duration of eating in your experiment.

<u>Mindful Eating Behaviour Practice - (MEBP; Mantzios, 2022)</u>

While you're enjoying your meal, keep these questions in mind. They're designed to guide your thoughts and feelings throughout your dining experience. Some questions might not be directly about the food, but they're included to encourage you to explore your thoughts and emotions during the meal. Feel free to have the questions in front of you without the need to write down your responses. Revisit them frequently to make the most of this reflective experience.

Let's begin by immersing ourselves in the act of eating. Dedicate your attention to the following three questions for the initial moments, allowing yourself ample time to explore the nuances of smell, taste, and texture. If any one of these aspects resonates more strongly with you and enhances your connection with your food, feel free to focus predominantly on that particular sense. While taste tends to be the primary focus for many, choose the one that captivates you the most.

Allocate a few minutes to this sensory exploration before proceeding to the remaining prompts.

How does it smell?

How is its texture?

How does it taste?

Now, let's shift the spotlight onto you! As you read each statement below, take a moment to reflect on your personal experience. Afterwards, circle back to the initial questions we discussed on sensory explorations such as taste or smell.

Feel free to consider the questions again after contemplating each statement.

I am having <u>thoughts</u> that are not allowing me to experience the food entirely. It is okay, that happens to everyone. Just tell yourself “I just had a thought”, and focus on the taste, texture and/or smell of the meal.

I am having <u>feelings</u> that are not allowing me to experience the food entirely. It is okay, that happens to everyone. Just tell yourself “I just had a feeling”, and refocus on the taste, texture and/or smell of the meal.

(<u>Note</u> that anything else, such as a sound, can equally distract you from paying attention to your meal. Every time your mind wanders off the meal to something else, aim to return to the questions on either taste, smell or texture.)
